# Making the most of audit and feedback to improve diabetes care: a qualitative study of the perspectives of Australian Diabetes Centres

**DOI:** 10.1186/s12913-022-07652-9

**Published:** 2022-02-24

**Authors:** Matthew Quigley, Sophia Zoungas, Edward Zimbudzi, Natalie Wischer, Sofianos Andrikopoulos, Sally E. Green

**Affiliations:** 1 School of Public Health and Preventive Medicine, Monash University, Melbourne, VIC 3004 Australia; 2 Diabetes and Vascular Medicine Unit, Monash Health, Clayton, VIC 3168 Australia; 3 National Association of Diabetes Centres, Sydney, NSW 2000 Australia; 4 Australian Diabetes Society, Sydney, NSW 2000 Australia

**Keywords:** Audit and feedback, Diabetes Mellitus Type 1, Diabetes Mellitus Type 2, Framework, Quality Improvement, Consolidated Framework for Implementation Research, CFIR, Qualitative

## Abstract

**Background:**

Diabetes has high burden on the health system and the individual, and many people living with diabetes struggle to optimally manage their condition. In Australia, people living with diabetes attend a mixture of primary, secondary and tertiary care centres. Many of these Diabetes Centres participate in the Australian National Diabetes Audit (ANDA), a quality improvement (QI) activity that collects clinical information (audit) and feeds back collated information to participating sites (feedback). Despite receiving this feedback, many process and care outcomes for Diabetes Centres continue to show room for improvement. The purpose of this qualitative study was to inform improvement of the ANDA feedback, identify the needs of those receiving feedback and elicit the barriers to and enablers of optimal feedback use.

**Methods:**

Semi-structured interviews were conducted with representatives of Australian Diabetes Centres, underpinned by the Consolidated Framework for Implementation Research (CFIR). De-identified transcripts were analysed thematically, underpinned by the domains and constructs of the CFIR.

**Results:**

Representatives from 14 Diabetes centres participated in this study, including a diverse range of staff typical of the Diabetes Centres who take part in ANDA. In general, participants wanted a shorter report with a more engaging, simplified data visualisation style. Identified barriers to use of feedback were time or resource constraints, as well as access to knowledge about how to use the data provided to inform the development of QI activities. Enablers included leadership engagement, peer mentoring and support, and external policy and incentives. Potential cointerventions to support use include exemplars from clinical change champions and peer leaders, and educational resources to help facilitate change.

**Conclusions:**

This qualitative study supported our contention that the format of ANDA feedback presentation can be improved. Healthcare professionals suggested actionable changes to current feedback to optimise engagement and potential implementation of QI activities. These results will inform redesign of the ANDA feedback to consider the needs and preferences of end users and to provide feedback and other supportive cointerventions to improve care, and so health outcomes for people with diabetes. A subsequent cluster randomised trial will enable us to evaluate the impact of these changes.

**Supplementary Information:**

The online version contains supplementary material available at 10.1186/s12913-022-07652-9.

## Background

The rising prevalence of Diabetes Mellitus (DM) is leading to poor health outcomes and higher healthcare burden [1, 2]. In 2017 it was estimated that 5 million deaths globally were due to diabetes and that total global health expenditure related to diabetes and its management was 850 billion US dollars [1, 2].

In Australia, people who live with diabetes attend a mixture of primary care settings, secondary health services such as community health centres, and outpatient clinics within tertiary hospitals. Many of these health settings are members of the National Association of Diabetes Centres (NADC), which helps guide and promote evidence-based policies and procedures in the management of diabetes [3]. In collaboration with the NADC, the Australian National Diabetes Audit – (ANDA) routinely collects information about clinical indicators (e.g. HbA1c, blood pressure and lipid levels) and health service indicators (e.g. proportion of patients receiving annual retinal examination) from Diabetes Centres. ANDA is an annual audit and feedback activity where de-identified cross-sectional patient data are collected from Diabetes Centres across Australia (audit), collated and compared, and then reported back to participating Diabetes Centres (feedback).

Benefits of large-scale auditing initiatives such as ANDA include the ability to show variation in practice between centres and to identify lack of concordance with evidence-based recommendations [4]. ANDA therefore aims to assist participating centres in using this feedback to inform quality improvement (QI) activities, with the ultimate aim of improving diabetes outcomes for their patients [5, 6]. Despite this potential, descriptive data from pooled ANDA reports shows room for improvement in key clinical measures including HbA1c, blood pressure and lipid levels, with little change over time, despite feedback [6–8]. This lack of change over time in key clinical outcomes was integral to the desire to improve the feedback delivered as part of ANDA. One approach would be to deliver a top-down redesign, where the research team redesigns the feedback based on beliefs about what might assist users. Another approach is to underpin the delivery of audit and feedback interventions with theory [9, 10].

The importance of tailoring audit and feedback by underpinning it with behaviour change or implementation theory is emphasised in the literature [11–17]. However, it can be challenging to operationalise theory to underpin development of specific audit and feedback interventions, and to apply these to specific contexts and user needs (tailoring) [13]. Many theories with overlapping constructs have been used in previous studies [13, 15, 18]; some focused on behavioural change at an individual level (e.g., individual clinicians), others on a system or organisational level. The Consolidated Framework for Implementation Research (CFIR) [19] brings together a number of theoretical constructs to overcome this, allowing interventions to be tailored to meet the needs of those enacting change, and to their context and settings [19]. Domains of the CFIR include characteristics of the intervention itself, constructs relevant to the inner and outer setting, characteristics of the individual and constructs relevant to the process [19].

Theory is invaluable in guiding development and implementation of audit and feedback interventions and guided us in this qualitative investigation regarding the use of ANDA feedback by Australian Diabetes Centres. We had hypothesised that the feedback currently provided may not be used as widely or enthusiastically as we would like, and that there may be design elements or cointerventions that would assist. In accordance with recommendations from the audit and feedback literature, we proposed a redesign of ANDA feedback potentially including shorter reports, different data visualisation techniques and infographics, a clinical dashboard with a traffic-light style interface, a report card style summary, and a pre-populated PowerPoint summary of the feedback, in addition to an additional cointervention such as coaching [4, 20, 21]. However, only qualitative research with participating diabetes centres could provide guidance on whether our hypothesis about current levels of use of ANDA feedback were correct, and whether our proposed feedback redesign and cointerventions were appropriate to the needs of end users. We are not aware of previous studies that have investigated the feedback needs of Diabetes Centres participating in a national clinical diabetes audit.

Harnessing the CFIR, this study addresses the need to better understand the way in which ANDA feedback is currently used by Diabetes Centres, the barriers and enablers to its use, and to explore enhanced, tailored, potentially more effective methods for delivering the ANDA feedback and supporting Diabetes Centres to use it. This will inform the development and delivery of a tailored, theoretically underpinned and enhanced ANDA feedback intervention aiming to improve key clinical indicators. This will be evaluated in a later randomised trial. Without undertaking a theory-based qualitative needs assessment, the interventions developed for the subsequent cluster randomised trial would likely be limited to pragmatic solutions that did not adequately meet the needs of end users [10].

### Aims

The overarching aim of this study was to understand the needs of end users in order to improve clinical practice and health outcomes for people living with diabetes through the design, delivery and evaluation of an audit and feedback intervention tailored to the needs of Diabetes Centres (audit and feedback users). To inform the development of this audit and feedback intervention, and identify potential co-interventions to support its implementation, this study aimed to elicit user perceptions of:

1. the utility and acceptability of feedback currently provided as part of ANDA
2. preferred options for redesign of the feedback
3. barriers to acting on the feedback in its current format
4. enablers to more effective use of feedback
5. any desired co-intervention with potential to support Diabetes Centres to engage with the feedback and use it to inform QI activities

## Methods

**Fig. 1:**
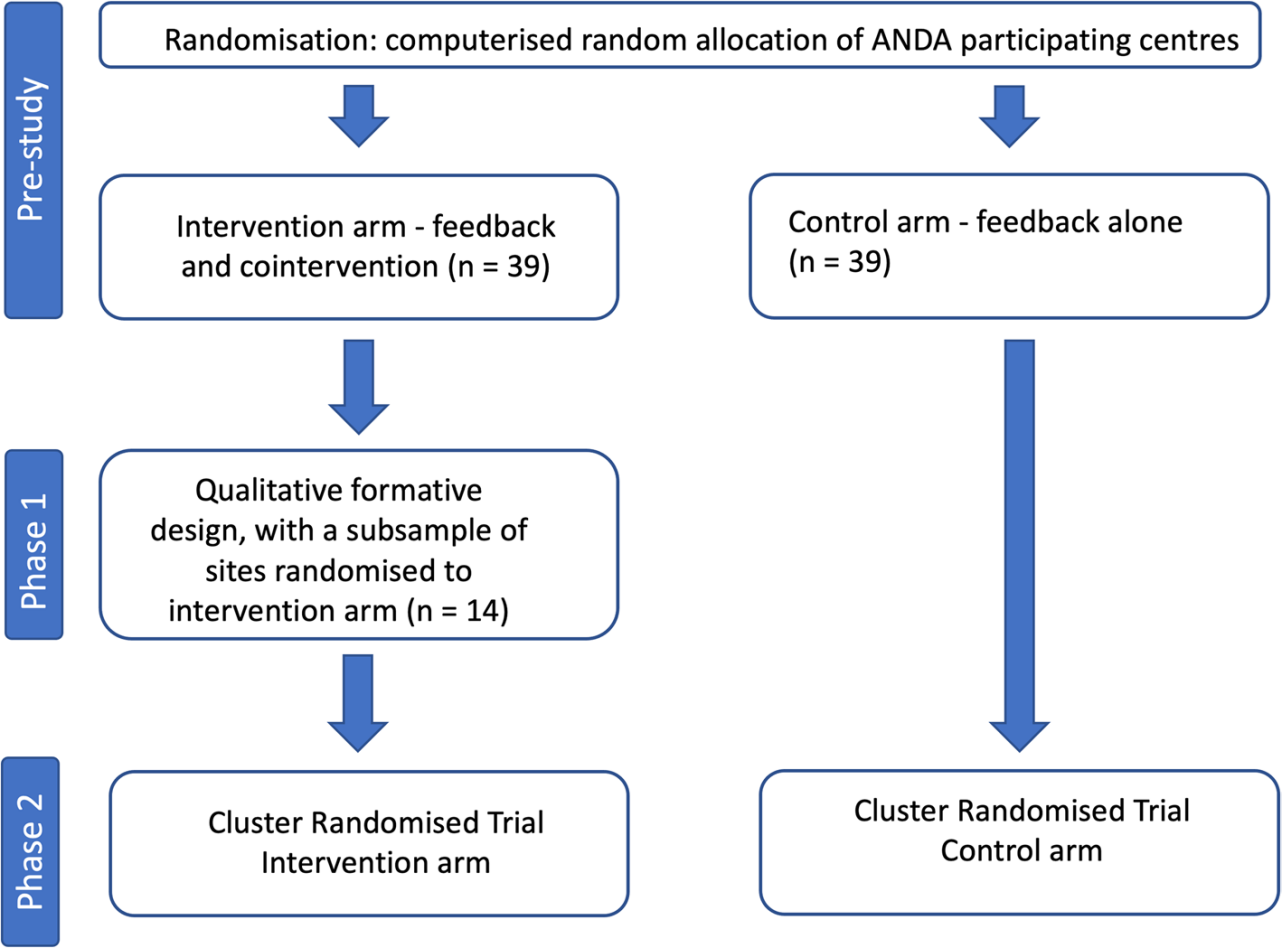
Study overview. Figure 1 shows the larger body of work within which this project sits. This manuscript describes Phase1, the qualitative formative work, which will inform the development of the intervention delivered in Phase 2

This qualitative study is Phase 1 of a larger project, as shown in Fig. 1.

A later cluster randomised trial (Phase 2) will evaluate the intervention to be developed in response to the results of this qualitative study. To avoid contamination of the trial control group, this qualitative study was conducted only in those centres that will receive the trial intervention. Randomisation therefore occurred prior to commencement (see Pre-Study Phase, Fig. 1).

### Recruitment

All centre leads and staff who receive and act on ANDA results in a Diabetes Centre randomised to the intervention arm of the later trial were eligible to participate. We included a range of participants representative of the Diabetes Centres who participate in ANDA (i.e. Diabetes Centre type (primary/secondary/tertiary and regional or metropolitan location) and staff roles (doctors, nurses, diabetes educators).

Formal invitations to participate were sent to the leads of eligible Diabetes Centres. Participation was voluntary. Potential participants who responded to the initial email were contacted by the project lead for participation and informed consent.

A Participant Information and Consent Form (PICF) detailing the exact nature of the study; what it involved for the participant; the implications and constraints of the protocol; and any risks involved in taking part, was sent to potential participants. Participants who replied and expressed interest in taking part were contacted by the project lead to organise an interview via video conferencing or telephone. Verbal consent was sought (confirmed at the beginning of each interview and defined within the interview schedule) and participants also returned a signed PICF via email. Participants had the right to withdraw from the study at any time, with no consequences for withdrawal. Sampling continued until thematic saturation was reached [18]. Human Ethics approval was granted by Monash Health HREC (Monash Health Ref: RES-19–0000-704L) and Monash University HREC (Ref: 22,618).

### Interviews

A semi-structured interview guide (Additional File 1) was developed to ensure all key topics were covered, including: (1) perceptions of the current format of feedback; (2) ideas for alternative formats; (3) barriers and facilitators to using the feedback to prioritise and facilitate quality improvement activities and (4) perceptions and preferences regarding a range of potential co-interventions that may accompany the feedback and support its use. The questions were underpinned by the CFIR and refined with subsequent interviews. The analysis was occurring concurrently with the interviews, leading to refinements to the interview guide as the research progressed.

Interviews were conducted by the project lead (MQ) via video conferencing using the platform Zoom [22], or by telephone. Most of the interviews were with single practitioners; for two metropolitan sites and one regional site, interviews were conducted with pairs of staff, due to their working relationship in regard to ANDA (i.e. shared responsibility or job share arrangements). Participants were also invited to contact the interviewer directly if there was further information they wished to share that was not covered in the interview. Interviews were recorded and professionally transcribed, then de-identified (coded) and stored on a secure University server.

### Analysis

De-identified transcripts were imported into NVIVO qualitative data analysis software [23] and analysed thematically, underpinned by the domains and constructs of the CFIR [19]. Participant responses were systematically mapped to the relevant domain of the CFIR, then to the appropriate constructs and sub-constructs. Common beliefs about the current ANDA feedback were identified and labelled as ‘belief statements’, as they arise from similar statements and reflect similar core beliefs [24–27]. We followed the approach of Francis et el., where qualitative data is mapped to constructs of a theoretical framework [25]. While Francis et al. used the Theoretical Domains Framework [28], we chose to use the CFIR as it is better suited to understanding problems with intervention acceptance, while the TDF is centred on investigating individual behaviour change [10, 19, 25, 28].

Our choice of thematic analysis as opposed to another qualitative analysis method was guided by other studies that have used theoretical frameworks to aid in data collection, in combination with thematic analysis to identify common themes pertaining to the research questions and theoretical frameworks [10, 25–27, 29–31]. Such an approach was appropriate as the purpose of this study was to investigate specific aspects of the participants use of ANDA feedback and desired changes to the feedback provided, to inform the development of interventions in the subsequent trial.

**Fig. 2:**
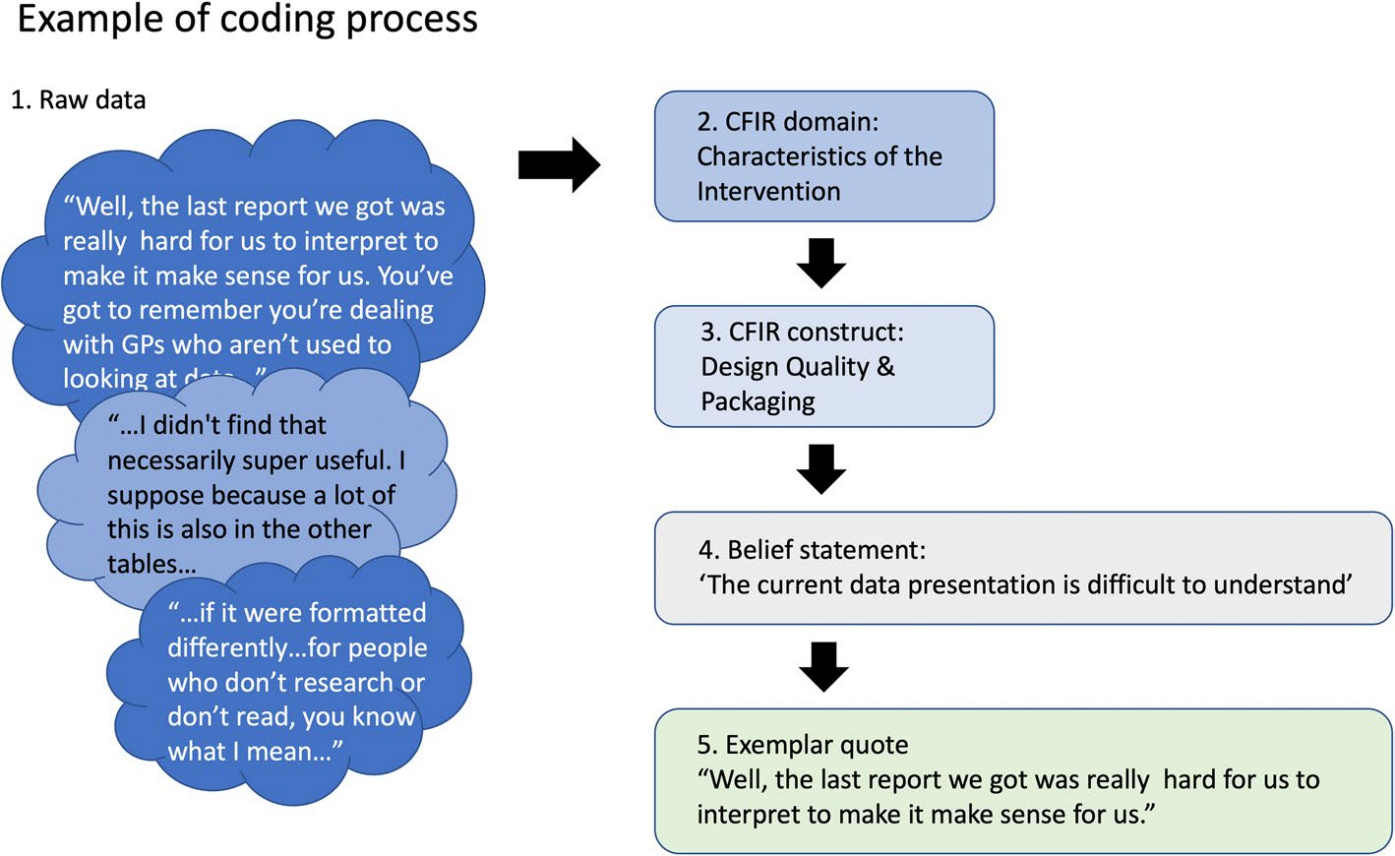
Example of coding process of mapping data to CFIR and eliciting belief statements. Figure 2 shows an example of the coding process. The raw data, represented in the bubbles on the left, is first mapped to the domains and constructs of the CFIR. Then, belief statements are generated that represent a synthesised version of participant comments reflecting common beliefs. After checking these belief statements against the raw data, exemplar quotes are chosen from the raw data

An illustrative overview of the coding and analysis process is provided in Fig. 2.

The analysis was managed by the project lead (MQ), with the assistance of two other investigators (SG, EZ) to address four phases of analysis: (1) building familiarity with the data through review of the transcripts; (2) building a codebook by mapping the transcripts to the domains, constructs and sub-constructs of the CFIR with preliminary codes, and identification of any data that did not directly map to the CFIR; (3) review of the preliminary codes and; (4) identification of belief statements and ensuring that data saturation had been reached [25, 26]. A subset of the transcripts was double coded by two of the authors (MQ, EZ) to ensure consistency, and throughout this coding phase the project team met regularly to support the analysis and discuss emerging themes.

The dataset supporting the conclusions of this article is included within the article (and its additional file(s)).

## Results

Forty Diabetes Centres (17 metropolitan and 23 regional) were invited to participate in the study. A total of 14 interviews (with 17 participants in total) were conducted between November 26, 2019 and January 14, 2020, after which there were no new emerging themes and saturation was considered to be reached. Interviews took between 30 and 75 min (mean interview duration: 50 min, 54 s). Participants represented a diverse range of healthcare professionals from Diabetes Centres involved in receiving and using ANDA feedback. Six participating centres were metropolitan centres and eight were regional centres.

A summary of the participant demographics and centre details is shown in Table 1.

**Table 1:** Participants

Participants		
**Centres invited to participate**		
	Metropolitan (*n* = 17)	Regional (*n* = 23)
**Centres who participated**		
	Metropolitan (*n* = 6)	Regional (*n* = 8)
**Type of centre**		
	Tertiary care (*n* = 6)	Primary care (*n* = 3)
		Secondary care (*n* = 1)
		Tertiary (*n* = 4)
**Participants**	4 m, 13 f	
	Metropolitan	Regional
Doctors/Endocrinologists	*n* = 4	*n* = 1
Nurse practitioners	*n* = 1	*n* = 2
Nurse manager	*n* = 1	
Clinical Nurse Consultant	*n* = 1	
Clinical Nurse Specialist	*n* = 1	
Dietician		*n* = 1
Credentialled Diabetes Educators		*n* = 4
Manager		*n* = 1

Table 1 shows the demographics of the participants. *n* = number, *m* = male, *f* = female

### Aim 1. To elicit user perceptions regarding the utility and acceptability of the feedback currently provided as part of ANDA

Several belief statements regarding the utility and acceptability of current ANDA feedback were identified. These beliefs mapped to the CFIR domain of ‘Intervention characteristics’ and the associated constructs of ‘Design Quality and Packaging’, ‘Complexity’, ‘’Evidence Strength and Quality’, ‘Cost’, and ‘Relative Advantage’. For clarity and consistency, CFIR domains and constructs/sub-constructs are presented in **bold**; belief statements are presented within inverted commas (' ') ; and direct quotes are presented in *italics with quotation marks (“ “).* Tables with full data are shown in Additional File 2.

**Design Quality and Packaging** was encapsulated in the belief statement ‘The current data presentation is difficult to understand’ and was reported by more participants from regional sites than metropolitan sites: *“You almost have to be a scientist to understand half those graphs. Not just anyone could look at those graphs and decipher, very quickly, how they relate to this service. You want all the data but just presented in a meaningful way.”* (Participant 2, regional).

Of those participants who did not find the presentation difficult to understand, some held the belief statement that ‘The current data presentation is useful but could be summarised for easier orientation’: *“I don’t think it’s too hard to read with the graphs and you can see at a glance how your institution is ranked. So, I think that’s useful. I don’t know how you do it differently. I suppose…some infographic in that you are in the top quartile for these indicators, bottom quartile for these indicators in some summary. That might make it easier to just work out which pages you want to look at more carefully.”* (Participant 9, metropolitan).

**Complexity** was raised by most participants from both regional and metropolitan sites, who believed: ‘The current report is too long to be useful’: *“If there was sort of a 10-page document, a few graphs, and something that was more achievable to read, I think we’d read the whole lot. [Our last report was] 150 pages, I was sort of skimming and just sort of floating through it. I haven’t even – to be honest, I haven’t read it.”* (Participant 4, regional).

**Evidence strength and quality** was reflected in the belief statement ‘The current benchmarking isn’t comparing like with like’, expressed by a similar proportion of regional and metropolitan participants: *“The populations in my observation are actually quite different and heterogeneous so benchmarking against others is probably one of the limitations of the ANDA reports because you’re not necessarily comparing like with like. But I certainly find it useful to benchmark with ourselves from year to year”* (Participant 7, metropolitan).

**Cost** was reflected in the participants’ belief that ‘The data collection period is burdensome’. This was more common among metropolitan participants but was also raised by participants from regional centres: *“… it is quite time consuming, if you want to do it properly that is, and it costs money. We’re lucky that we’ve got some funding that we can pay someone to do that… The last two years we’ve funded her to do, I think, about 15 days of work just completing audits”* (Participant 2, regional).

**Relative advantage** was reflected in the belief statement ‘We value the ANDA report as a measure of our clinical practice’. This belief was more predominant among metropolitan sites: *“In our current state the ANDA survey is our only way of collecting clinical data…So to us, ANDA is critical at this stage, to support a process of collecting some data around what we’re doing in our clinics”* (Participant 14, metropolitan).

### Aim 2. To elicit user perceptions regarding preferred options for redesign of feedback format

Perceptions were sought regarding options for redesigned feedback, including shorter reports, different data visualisation techniques and infographics, a clinical dashboard with a traffic-light style interface, a report card style summary, and a pre-populated PowerPoint summary of the feedback. Belief statements about these techniques mapped to the CFIR domain **Characteristics of the intervention** and construct of **Design quality and packaging**. A full list of belief statements with illustrative quotes are shown in (Additional File 2).

Reflecting the belief against Aim 1 regarding the need for a summary, most participants believed ‘A shorter report would be more engaging’. Some participants, particularly those in metropolitan centres, reported that also maintaining access to the full report is helpful, but all agreed that there would likely be more team engagement with a shorter report. Having the full data available as an electronic addition to summary data was an attractive option: *“I think that’s sensible and I think it makes people—I mean it’ll make people read the document more if it’s 20 pages but then when you need the additional data to make a statement, you can just print out the other 60 pages or whatever as well. I think it is useful to separate the two.”* (Participant 5, metropolitan).

A range of views were expressed regarding data visualisation techniques, but there were clear preferences. The belief statement ‘Infographics are a helpful data visualisation technique’ was shared by almost all participants, particularly in terms of clearly expressing summary data and as a way to disseminate audit feedback to a wider audience: *“Boom. You can see it, in summary. Bang.”* (Participant 4, regional).

The majority of participants were also positive about the use of a clinical dashboard (‘A dashboard is a helpful data visualisation technique’). While participants had varying views of the utility of traffic-light dashboards, most felt that a dashboard interface would be useful to prioritise discrete areas of clinical care, or to identify trends over time. *“”It’s very visual and I think if you were—if we were trying to explain where the data was to people that need to get that message across clearly, whether we’re pushing how well we’re doing and saying where all the green bits are or if we’re pushing an agenda to say we need to do better or we need more help and pushing the red parts of the dashboard, that would be helpful. I mean it is really, really easy to understand so I think that would be very helpful.”* Participant 5, metropolitan.

Commonly, participants expressed doubt about the utility of a report card style summary (belief statement ‘A report card may not be helpful in illustrating actionable clinical improvements’), as there were concerns about the appropriateness of the messaging (e.g. ‘needs improvement’), staff engagement and whether this format would provide actionable items: *“It, again, just seems like, “Oh yeah, they’re just telling us where we need to be headed.” I don’t think the format of that – because they are just statements. It might be okay as the front page of a report, as a summary in something, but in terms of individual staff, in a service, taking notice of it, I don’t think they’d look at it”* (Participant 12, regional).

All participants shared the belief statement ‘A pre-populated PowerPoint deck would be helpful for disseminating the data’. The succinct nature of this option was particularly favoured. *“I think a PowerPoint presentation would be great because we could do that as a team and like I said we don’t want a detailed report that details every item of the audit, we just want the key points basically.”* (Participant 8, metropolitan).

### Aim 3. To elicit user perceptions regarding the barriers to implementing feedback in its current format

Participants identified a range of barriers to implementing the feedback in its current form which mapped to the CFIR domain of **Inner setting** including constructs of **Implementation climate** and **Readiness for implementation**. Full details of the barriers and their relevant constructs and subconstructs are included in Additional File 2.

A subconstruct of **Implementation climate** is **compatibility**, relevant to participants’ commonly voiced concerns that the demands of the clinical environment impede clinical staff engaging with the audit and feedback. We summarise these many pressures in the belief statement ‘There’s a lack of engagement with the audit because of clinical pressure’. An example of this can be seen in the quote: *“I think people, possibly, aren’t as invested in it as we would like them to be. Because, the way the audits occur we’re funding a nurse, who doesn’t normally work with those clients [to collect] the data, so they’re [clinical staff] not really invested right from the start… they know the audits are happening, they know it’s their clients that are being audited, but they’re not really involved in an active way, from the beginning. So, to them, I think, they just possibly see it as, “Oh, another thing I’ve got to add to my list of jobs to do.””* (Participant 2, regional).

Another sub-construct of **Implementation Climate** is **relative priority**. The majority of participants believed that ‘Reviewing ANDA feedback and utilising it for developing QI activities is a low priority’. Although this was more commonly expressed in centres with smaller teams, similar ideas were generated by metropolitan and regional sites: “*Well we would say that we technically probably haven’t sat down as a team and gone through it as such… One, because a time factor, and two, because we’ve never worked out how to utilise the document to – I don’t know – use to our advantage so to speak.”* (Participant 4, regional).

Mapping to the construct of **readiness for implementation**, and more specifically the sub-construct of **access to knowledge and information**, some participants expressed the belief statement ‘Not everyone knows how to use the feedback to inform QI activities’. *“I think the report in itself, we know what’s going wrong. It’s actually having the means to address it and time is often a big issue, but sometimes it’s just the know-how”* (Participant 10, regional).

Mapped to the subconstruct of **Available resources**, the majority of participants (metropolitan and regional) believed ‘We have limited resources for implementation’. *“The biggest one is time to be honest and I guess along with that is the resources. In any public hospital the focus is on service provision, the delivery of clinical care. Although QI obviously has a flow on effect for that if you’re out on the ward all the time or in the clinic all the time or don’t have the admin [administration] support or the IT [information technology] support that is an obstacle to QI.”* (Participant 7, metropolitan).

Another barrier to implementation, aligned with the subconstruct of **Leadership engagement**, was the belief that ‘Lack of leadership engagement is a barrier to implementation’: *“…it could be useful if we had the right platform, and I mean like a committee or something that was dedicated to actually analysing the data and influencing some quality improvement activity. I have to take time to wade through the information, currently, and then present it in a form that management are, actually, going to take notice of.”* (Participant 2, regional).

### Aim 4. To elicit user perceptions regarding the enablers to more effective use of feedback

The participants identified a range of enablers to more effective use of audit feedback. These enablers related to different CFIR constructs, including **Inner setting** (sub-constructs: **readiness for implementation; leadership engagement**), **Implementation process** (sub-constructs: **Engaging: Champions and External change agents**) and **Outer setting** (sub-construct: **External Policy & Incentives**), as shown in Additional File 2.

Some participants identified that they were able to use the ANDA data to leverage leadership engagement and facilitate more effective use of audit feedback to help expand services to meet the needs identified in the audit. These perspectives contribute to the belief statement ‘We can use the data in ANDA feedback to engage leadership’, as demonstrated in the following quote: *“I think it’s actually because it is benchmarked that, really, time managers in our organisation take note. I would hate to see that removed from the audit and the report, because that’s really speaking executive management language, and it’s incredibly powerful, so just from a, I guess, purely a political point of view, I would just hate to not have that benchmarking. Because we really use that as leverage if…we have the lowest uptake of something, that’s incredibly important for us.”* (Participant 5, metropolitan).

Some participants believed that success stories from clinical champions were, or could be, enablers in the implementation process. ‘Success stories and the experiences of other centres are enablers’. A range of regional and metropolitan sites reported that hearing those stories from clinical champions who have successfully made change in their own centres inspires changes to build QI interventions at a local level. *“But also having perhaps one of the organisations that are doing it well, or having a call out to those that are meeting the needs and how are they addressing it, how are they achieving that result?… there are little things like just being aware of where the services and how to access those services, especially in an ever-changing population, there are things that I think we can learn from other places that are doing it well.”* (Participant 10, regional).

A similar proportion held the belief that ‘Mentoring from other centres would be an effective enabler of implementation’. This was particularly true for regional centres: *“I’m sure there’s not only one centre that has the same issues. It would be good to work together and if there’s not a solution, work together and try and address it in the same way so that we can see those outcomes are working or – I hate this reinventing the wheel all the time, that round Australia five centres might have the same issue but we’re all trying to fix it with our own little committees and things rather than if someone’s got the answer, why not share it?”* (Participant 10, regional).

This belief extended to the value of peer-to-peer support (‘Peer to peer support is an enabler’). Participants talked about their desire for peer support and its value. *“I went along and networking with other services and seeing all of these amazing QIs and amazing things that other centres were doing with less or more resources or less or more staff and also sharing some of the challenges that we all face, that was probably the first motivating factor.”* P 7, metropolitan.

Several participants felt that ‘External policy and incentives can be effective enablers’. Examples such as NADC accreditation or Professional Development programs in professional societies can be effective enablers to engage staff in the process of audit and feedback: *“When we last had our…accreditation…the fact that we’d participated in ANDA was an incredibly important element of our quality improvement. We had a lot of evidence to show that we’d done good quality improvement.”* P 11, regional.

### Aim 5. To elicit user perceptions regarding co-intervention that is likely to support implementation of feedback and development of QI activities

A number of options for additional interventions to support feedback and facilitate quality improvement activities were explored with participants, with focus on their feasibility and acceptability. These ideas did not contribute to belief statements but explored the potential of not only improving the format of the feedback, but also supplementing it with a supporting co-intervention. Participants from both regional and metropolitan sites had suggestions for co-interventions.

Coaching: there were concerns regarding the logistics of providing coaching: *“… we are very time poor, but it could be good if someone could go this is where you’re doing well, this is what you need to improve on, I think that would be useful, but it would need to be straight to the point.”* (Participant 8, metropolitan). Many of the participants offered that with improved feedback, coaching should not be necessary: *“I would hope that it was presented in a way that you could, easily – like you wouldn’t need coaching if it was clear how to interpret it.”* (Participant 2, regional).

Change champions: Webinars or online delivery of stories from change champions working in exemplary sites were considered: *“So, if something’s been successful, share it. I know people do that at conferences and things, but not everyone can get to those, and it’s not specifically about this data. So, if there was some sort of showcase facility, on the website, that specifically related to quality improvement activities that can come from such a report, that would be useful.”* (Participant 12, regional).

Educational activity: instructional webinars to help sites in data collection or to understand the feedback were suggested *“I think possibly if there [were] more webinars around it [ANDA] where people could access around—again maybe a webinar for first timers using it, a webinar around some of the tricks of getting the best data and what have you, understanding the reports. I think if there [were] webinars that were available to look at any time for members participating, I think that would be incredibly helpful.”* (Participant 5, metropolitan).

Community of practice: reflecting the diversity of sites, participants wanted examples of QI interventions from similar sites to theirs. Commonly, this was an issue for participants from regional sites, but was also relevant for metropolitan sites: *“I’d like to get some ideas about perhaps how other institutions perhaps similar to mine with similar funding issues, similar problems, have tried to resolve the issues that they had… it would be probably something similar to a website, I suppose, where people can post something, this was the problem, this is how we solved it or tried to solve it and we actually found usefulness from that. So, I think, yeah, it could be useful for us to get some ideas.”* (Participant 9, metropolitan).

## Discussion

This study aimed to elicit user perceptions of: (i) the utility and acceptability of feedback currently provided as part of ANDA; (ii) preferred options for redesign of the feedback; (iii) barriers to acting on the feedback in its current format, (iv) the enablers to more effective use of feedback and (v) any desired co-intervention with potential to support diabetes centres to engage with the feedback and use it to inform QI activities. Following interviews with representatives of 14 Australian diabetes centres, data was analysed thematically, guided by the CFIR.

ANDA audit and feedback is intended to improve health outcomes for people with diabetes [6], reduce variation in clinical management and facilitate evidence-based practices. In order to achieve these goals, feedback should meet the needs of the people it is intended to support. Our results will inform the redesign of ANDA feedback, and have potential to inform other audit and feedback initiatives.

We found participants wanted a shorter report with a more engaging, simplified data visualisation style. Identified barriers to use of feedback were time or resource constraints, as well as access and knowledge about how to use the data provided to inform the development of QI activities. Perceived enablers included leadership engagement, peer mentoring and support, and external policy and incentives. Potential cointerventions to support use are exemplars from clinical change champions and peer leaders, and educational resources to help facilitate change. These results supported our contention that the existing feedback may not be used as widely or enthusiastically as we would like it to be.

### A shorter, simplified, more visual report

The need for a short, visual report with clear key messages is consistent with the established barrier to clinician use of audit and feedback: challenges in identifying key messages from the data provided [21]. This may be explained by cognitive load theory, an influential theory in educational design literature, which, in part, explains that providing complex information in large amounts may make it more difficult to understand key messages [32–34]. Consistent with this theory, and recommendations from recent audit and feedback design literature, we will redesign the ANDA feedback to present data about key outcomes in a more intuitive data visualisation without extraneous elements. This may help reduce distraction from the key messages [20, 21, 35, 36].

### Knowledge to support action

A Cochrane review and a recent meta-analysis of qualitative studies reveal common barriers to audit and feedback use including a perceived lack of knowledge about how to act on the feedback [4, 21]. Although the time and resource constraints of individual Diabetes Centres also impede investment in QI activities and are beyond the scope of ANDA, there is potential to provide educational resources to Diabetes Centres. Such resources might include guides to help identify patterns in the data, and exemplar action plans for developing QI activities from the data.

### Support use of ANDA feedback in quality improvement

While some of the perceived enablers of feedback use were outside the scope of ANDA (e.g., external policy and incentives), there were actionable enablers to support feedback use (such as PowerPoint presentations to facilitate dissemination within Diabetes Centres) and suggested cointerventions such as mentoring from peers and stories from change champions, delivered through a community of practice. Investment in establishing these mechanisms may allow participants to feel more empowered in developing QI programs and help to reduce unnecessary duplication at a local centre level [4, 20, 35, 37].

### Strengths and limitations of this study

The strengths of this study included underpinning the interviews and analysis with a well-established conceptual framework. This enabled systematic, facilitated thematic analysis with focus on factors most likely to be relevant/known to be relevant from organisational theory[19]. As such, this work builds on the work of others who have designed and used the CFIR [19, 38–40]. Our study population was recruited from a variety of settings including metropolitan and regional as well as primary, secondary and tertiary care to allow generalisability of our study findings. The study was conducted in partnership with the ANDA team and the participating Diabetes Centres, so it is likely to have greater uptake and relevance to clinical practice. As these results will inform future work to optimise an existing audit and feedback activity, there is potential to positively impact the care of people with diabetes.

Study limitations include a small sample size with more participants from regional centres. Recruitment was impeded as capacity to participate was difficult due to time constraints for clinicians in busy Diabetes Centres. Our sample size was limited to sites randomised to the intervention group in the subsequent trial, in order to avoid contamination of the control group in the trial. While different health care professionals were participants, our questions were related to their common experience and use of ANDA feedback. Despite the small sample size, in keeping with the concept of data saturation, by the 14^th^ interview there was no new information emerging from the interviews [41, 42]

In addition, we did not double code the transcripts of all interviews. However, a random subset was double coded, and investigators met frequently to discuss the coding, analysis and interpretation of results. Our study was conducted in a very specific context, to inform intervention development within an existing audit and feedback activity, however our findings are consistent with those of others, and may contribute to the growing body of knowledge informing effective ways to deliver feedback.

All authors are involved in aspects of diabetes research, which may be perceived as a limitation by some. The project lead (MQ) was also the interviewer and the lead data analyst. He is a PhD student working with the ANDA team to optimise the feedback provided as part of the ANDA clinical audit. He also lives with type 1 diabetes and therefore has an interest in improving healthcare outcomes for people living with diabetes. The secondary analyst and co-author (EZ) is a diabetes healthcare professional with an established record of diabetes related research. The senior author (SG) is an established researcher with a wide variety of work, including work on the Australian Living Evidence Guidelines in Diabetes [43]. The review of the data and interpretation by these different authors helped to ensure that the risk of individual bias was low in the interpretation of the data. The other authors (SZ, NW and SA) are all experienced diabetes clinicians and authors who are involved in ANDA but were not directly involved in coding or analysis of the data.

None of the authors have previous experience in the development of audit and feedback interventions, nor any conflict of interest regarding the proposed interventions about which opinions were sought from the participants in this study.

### Future directions for ANDA feedback

Research evaluating audit and feedback provides some insight into feedback interventions that are effective in facilitating clinical change [4, 20, 44, 45]. Including more than one form of feedback; feedback that is provided in both a written and verbal format; and feedback which offers explicit goals and a specific action plan can result in better outcomes [4, 46]. Graphical elements such as funnel plots and infographics may also assist with transmitting complex information in an intuitive manner [12, 47, 48].

Drawing on this body of knowledge and informed by our findings, we intend to redesign ANDA feedback to reduce the volume of messages provided and to simplify the data visualisation. We hypothesise that Diabetes Centres will find the redesigned feedback easier to interpret and to incorporate into clinical practice through QI activities. We will evaluate this in a cluster randomised trial to assess acceptability, utility and impact on selected clinical outcomes. We will also introduce and evaluate a cointervention in the form of educational resources and peer support for acting on feedback and undertaking QI activities, as these concepts were clearly and consistently raised by participants.

## Conclusion

This qualitative study supported our contention that the format of ANDA feedback presentation can be improved. Healthcare professionals suggested actionable changes to current feedback to help optimise engagement and potential implementation of QI activities. These suggestions offer a valuable opportunity to inform redesign of the ANDA feedback, to consider the needs and preferences of end users and to provide feedback and other supportive cointerventions to improve care and so health outcomes for people with diabetes. A subsequent clinical trial will enable us to evaluate the impact of these changes.

## Supplementary Information

**Additional file 1.**

**Additional file 2.**

## Data Availability

The dataset supporting the conclusions of this article is included within the article (and its additional files).

## Abbreviations

ANDA: Australian National Diabetes Audit
QI: Quality improvement
CFIR: Consolidated Framework for Implementation Research
A&F: Audit and feedback
DM: Diabetes mellitus
US: United States
NADC: National Association of Diabetes Centres
PICF: Participant Information and Consent Form
HREC: Human Research Ethics Committee
MQ: Matthew Quigley
IT: Information technology
