# Supplementary material for: Making the most of audit and feedback to improve diabetes care: a qualitative study of the perspectives of Australian Diabetes Centres
Source: BMC Health Serv Res. 2022 Feb 24;22:255. doi: 10.1186/s12913-022-07652-9 (PMC8876070; doi:10.1186/s12913-022-07652-9)
Supplement: Supplementary file 1 — Additional file 1. [file 12913_2022_7652_MOESM1_ESM.docx]

**Additional File 1.**

**Semi-structured interview guide**

**Note: Questions evolved as the interviews were being conducted. This is an indicative guide only. Questions are framed using the Consolidated Framework for Implementation Research (CFIR).**

**Purpose**

The purpose of this interview is to enable the researchers to better understand the use of diabetes audit feedback in clinical practice, but the performance of individual clinicians or diabetes centres is not being evaluated. Furthermore, there is no linkage of the qualitative data collected in this study with the clinical or process outcomes collected as part of ANDA, nor linkage of the qualitative data collected in this study with individual clinicans or site ID.

**Consent**

Do you consent to participate in this interview?

**Questions related to study objectives**

| **Study objective** | **Question** | **CRIR construct*** |
| --- | --- | --- |
| **Elicit stakeholder perceptions regarding the utility and acceptability of current ANDA-AQCA feedback format** |  |  |
|  | Are you familiar with the site report delivered to your site as part of ANDA? (Show example) | **1** |
|  | Overall, how useful are these reports to your site? | **1** |
|  | Could you elaborate on this? | **1** |
|  | Is the data presented in a way that you find helpful, or easy to understand? | **1** |
|  | Is the process of interpreting the data to other people in your organisation intuitive? | **2** |
|  | How helpful is the data for translation to people outside your site? For example, if you are making a funding request to implement a quality improvement activity? | **3** |
|  | How acceptable is the current format of the site report to your site? Does it meet your needs? | **1** |
|  | How do you feel about the length of the report? | **1** |
|  | How does your site use the data presented in the site report? | **4** |
|  | Does your site use the data in the site report to inform quality improvement activities? | **5** |
|  | What kinds of quality improvement activities have you used in the past, based on the data in the site report? | **5** |
|  | How helpful are the explanatory notes? (show example) | **1** |
|  | How helpful is the 3-year comparative data? (show example) | **1** |
|  | How helpful is the frequency count data? (show example) | **1** |
|  | How helpful is the mean/descriptive data? (show example) | 1 |
|  | How helpful is it to have the missing data shown? (show example) | 1 |
|  | How helpful are the benchmarking graphs and tables? (show examples) | 1 |
|  | How many people at your site are involved in review of the site reports? | 2 |
|  | What kinds of roles/professions are these people? | 2 |
|  | Are all these people involved in trying to implement quality improvement activities? | 4 |
|  | Do you have a structured development/implementation process for quality improvement activities arising from the ANDA reports? | 5 |
|  | Could you tell me about this process? | 5 |
|  | Is there anything else that you’d like to add about the reports that you currently receive? |  |
| **Elicit stakeholder perceptions regarding preferred options for redesign of feedback format** |  |  |
|  | If you could have your ideal version of the report your site receives, what would it look like? Can you describe this for me? | 1 |
|  | How much data would you like to see about your site? Follow-up: what kind of data? | 1 |
|  | Are there other ways that you would prefer to see the data presented, compared to the current format (such as different graphs etc)? | 1 |
|  | Would something like a dashboard help? (show example from 2018 ANDA-AQSMA site report) | 1 |
|  | Would you be interested in something like a report card, which describes the areas your site is achieving and the areas where there is room for improvement? (show example) | 1 |
|  | What would the ideal length of the report be for you? | 1 |
|  | How would you feel if we gave you a much shorter version of the report – for example, 25 pages or so? (show example of another registry report) | 1 |
|  | What would be the essential elements that you would need in a shorter report? | 1 |
|  | How would you feel about information presented in infographics? (show example) | 1 |
|  | Would it help if we gave your site a pre-populated PowerPoint slide deck with your ANDA data, so that you could use it in meetings within your site and across different disciplines and in presentations to people outside your organisation? | 1/2/3/4/5 |
|  | If so, what do you think this would need to include? | 1 |
|  | Who would the slide deck be prepared for? | 2/4 |
|  | How do you think your colleagues would feel about the alternatives that we have discussed? | 2 |
|  | Do you think they would be receptive to change? | 2 |
|  | Do you think that changing the format of the report would help you to develop QI activities from the ANDA data for your site? | 4/5 |
|  | Do you have other suggestions for changes that you would like to see in the presentation of the report? |  |
|  |  |  |
| **Elicit stakeholder perceptions regarding the barriers to implementing feedback currently provided and the enablers to more effective use of feedback** |  |  |
|  | Are there factors that make it difficult to implement quality improvement activities based on ANDA site report data? | 1/2/3/4/5 |
|  | Could you elaborate? For example, is it something about the report itself, or some kind of administrative or other issue in your organisation, or more related to external circumstances, such as the broader health organisation? | 1/2/3/4/5 |
|  | Do you ever feel that the case mix that you have at your site makes it too hard to achieve change? | 2 |
|  | Could you elaborate about this? |  |
|  | We have talked about factors that make it difficult to implement quality improvement activities based on ANDA site report data. Are there also factors that make it easier to implement these kinds of activities? | 2/3/4/5 |
|  | Could you elaborate about this? |  |
|  | Do you ever see colleagues from another centre present about a quality improvement activity and think “we could do that too”, or “well, we could never do that, because xxx” | 2/3/5 |
|  | What do you think might influence or impact on your ability to implement these kinds of activities? | 5 |
| **Elicit stakeholder perceptions regarding desired cointervention that is likely to support implementation of feedback and development of QI activities** |  |  |
|  | Apart from the report itself, are there other things that you think might help support implementation of feedback and development of QI activities? | 1/2/4/5 |
|  | Do you have ideas about what these might be? |  |
|  | How would you feel about having some kind of individualised coaching, or structured presentation to help interpret the site report? | 1/2/4/5 |
|  | Who would be the best person to do this coaching? For example, would it make a difference if it was a clinician or a researcher doing the coaching or presentation? | 1/2/4/5 |
|  | What about an electronic reminder to review the report? | 1 |
|  | If so, who would receive the reminder? | 2 |
|  | Would it need to be repeated? At what kind of frequency? | 2/4/5 |
|  | Would some kind of process-review proforma help (for example, to identify areas for improvement and suggested steps for action)? | 1 |
|  | Is there anything that we haven’t talked about that you think might be helpful to support implementation of feedback and development of QI activities? |  |
|  | Is there anything else you would like to add? |  |

* 1= Characteristics of Intervention, 2 = Inner setting, 3 = Outer setting, 4 = Individuals involved with intervention / Implementation process, 5 = Implementation process

**Closing**

Thank you very much for talking with me today. Your input is very valuable to this project and I really appreciate the time you’ve taken to talk with me. I’m obligated to inform you that any examples of feedback redesign and/or cointervention are examples only and that while development will be informed by stakeholder input, there is no guarantee that your preferred option will be what is delivered as part of the feedback redesign and/or cointervention.

If you have any further questions, please contact me.
