# Supplementary material for: Making the most of audit and feedback to improve diabetes care: a qualitative study of the perspectives of Australian Diabetes Centres
Source: BMC Health Serv Res. 2022 Feb 24;22:255. doi: 10.1186/s12913-022-07652-9 (PMC8876070; doi:10.1186/s12913-022-07652-9)
Supplement: Supplementary file 2 — Additional file 2. [file 12913_2022_7652_MOESM2_ESM.docx]

**Additional File 2. Tables of results aims 1 – 5**

**Table 1. Belief statements relevant to Aim 1**

| **Aim 1: To elicit user perceptions regarding the utility and acceptability of the feedback currently provided as part of ANDA** | | | |
| --- | --- | --- | --- |
| **CFIR domain/subdomain** | **Belief statement** | **Illustrative quote** | **Coverage** |
| Characteristics of intervention  Design quality and packaging | “The current data presentation is difficult to understand” | “You almost have to be a scientist to understand half those graphs. Not just anyone could look at those graphs and decipher, very quickly, how they relate to this service. You want all the data but just presented in a meaningful way.” (P 2, regional)  “Well, the last report we got was really hard for us to interpret to make it make sense for us” (P 11, regional)  “I found it helpful, and no, it’s not easy to understand. If you want it looked at by the wider team, no one’s looked at it other than me.” (P 6, regional) | 43% of total sites  75% of regional  0 % of metropolitan |
|  | “The current data presentation is useful but could be summarised for easier orientation” | “I don't think it's too hard to read with the graphs and you can see at a glance how your institution is ranked. So, I think that's useful. I don't know how you do it differently. I suppose you've had some infographic in that you are in the top quartile for these indicators, bottom quartile for these indicators in some summary. That might make it easier to just work out which pages you want to look at more carefully.” (P 9, metropolitan)  “I generally like the way that it’s presented…I do like how the report itself is very self-explanatory. I like how it’s broken into your site report at a glance and then the benchmarking report, the historical comparison.” (P 7, metropolitan) | 14% of total sites  0% of regional  33% of metropolitan |
| Characteristics of intervention  - Complexity  Sub-topic: length of report | “The current report is too long to be useful” | “If there was sort of a 10-page document, a few graphs, and something that was about more achievable to read, I think we’d read the whole lot. 150 pages, I was sort of skimming and just sort of floating through it. I haven’t even – to be honest, I haven’t read it.” (P 4, regional)  “It does become a little bit long, admittedly. Our last report was 200 pages…I must admit I don’t end up going through the whole thing.”  (P 14, metropolitan)  “I think if there was a shorter report and rather than sending the…150-page document to my team…if there was a 20-page summary of our ANDA for your perusal you’d probably get a bit more buy-in from more of the team members I think.” (P 7, metropolitan) | 93% of total sites  100% of regional  83% of metropolitan |
| Characteristics of intervention  - Evidence Strength & Quality | “The current benchmarking isn’t comparing like with like” | “The populations in my observation are actually quite different and heterogeneous so benchmarking against others is probably one of the limitations of the ANDA reports because you’re not necessarily comparing like with like. But I certainly find it useful to benchmark with ourselves from year to year.” (P 7, metropolitan)  “… in terms of the useability of the data, again it's just difficult to compare institutions sometimes when they aren't apples and apples, they're apples and oranges and pears or whatever”  (P 9, metropolitan)  “I mean [benchmarking] that is useful, it’s nice with those graphs to see where we fit with other sites, but like I said before, we’re comparing tertiary and secondary sites, which isn’t helpful.” (P 12, regional) | 54% of total sites  57% of regional  50% of metropolitan |
| Characteristics of intervention  - Cost | “The data collection period is burdensome” | “So yeah, it is quite time consuming, if you want to do it properly that is, and it costs money. We’re lucky that we’ve got some funding that we can pay someone to do that… The last two years we’ve funded her to do, I think, about 15 days of work just completing audits”  (P 2, regional)  “The reality is we’ve had to get some extra nursing time to help us do the audit for that month, to then help fill in the boxes and chase up results. So, it does, admittedly, have an imposition on the clinic” (P 14, metropolitan)  “I’m going to be completely honest and say that we’ve been doing this since 2014, and every year we have to make a decision, “Do we actually want to keep going with this, because what are we doing with the data?”” P 2, regional | 50% of total sites  43% 0f regional,  66% of metropolitan |
| Characteristics of intervention  - Relative advantage | “We value the ANDA report as a measure of our clinical practice” | “In our current state the ANDA survey is our only way of collecting clinical data…So to us, ANDA is critical at this stage, to support a process of collecting some data around what we’re doing in our clinics” (P 14, metropolitan)  “I’m just so grateful to have the tool to be able to use and I think people that are doing the audits should be looking at it as not just a task but really an important strategy for them to really grow and develop their service, but also to acknowledge the work that they do or the improvements that they’ve made. I think it really needs that promotion of it’s not just doing a task, it’s actually a pivotal part of growing your service, developing the service.” (P 5, metropolitan)  “We found that the reports have been helpful, particularly in the latter years when we’ve had more of a high participation rate…what we get back is the reward at the end of all of that, so we’re just very grateful for what we do get, given that we wouldn’t be able to do anything like this ourselves.” (P 12, regional) | 43% of total sites  25% of regional,  66% of metropolitan |

**Table 2. Belief statements relevant to Aim 2**

| **Aim 2: To elicit user perceptions regarding preferred options for redesign of feedback format** | | | |
| --- | --- | --- | --- |
| **CFIR domain & subdomain** | **Belief statement** | **Illustrative quotes** | **Coverage** |
| Domain: Characteristics of intervention  Subdomain: design quality and packaging  Option: shorter summary report | “A shorter report would be more engaging” | “I guess that would be useful, easier to distribute amongst the team, and also to go prepared if we needed to have discussions with executive. So, a shorter sort of overview summary, I think, would be useful, that was just the essential criteria data.” P 14, metropolitan  “I think that’s sensible and I think it makes people - I mean it’ll make people read the document more if it’s 20 pages but then when you need the additional data to make a statement, you can just print out the other 60 pages or whatever as well. I think it is useful to separate the two.” P5, metropolitan  “If we had something that was short and snappy that’s not going to take hours to pore over, then yeah, I think that would be really good. We’d definitely use that.” P 8, metropolitan | 79% of total  100% of metropolitan  63% of regional |
| Domain: Characteristics of intervention  Sub-domain: design quality and packaging  Option: data visualisation in infographics | “Infographics are a helpful data visualisation technique” | “Boom. You can see it, in summary. Bang.” P 4, regional  “Look this would be great, particularly then if it also came in a PowerPoint, for example, or a Word, that again you could easily put that in your own report if you were putting that to managers or in a presentation. I mean this sort of stuff is fantastic for PowerPoint presentations so I’d really welcome access to something like this.” P 5, metropolitan  “I think if we were presenting the data to the wider team following an audit, that sort of information could be useful.” P 13, metropolitan | 86% total  83% of metropolitan, 88% of regional |
| Domain: Characteristics of intervention  Sub-domain: design quality and packaging  Option: data visualisation in a dashboard | “A dashboard is a helpful data visualisation technique” | “I actually do find it useful to have the traffic light system for me. It does stand out so at least maybe some components of the report that actually do highlight areas that are critical and that do need attention and direct attention for future projects as well for service improvement.” P 13, metropolitan  “Yes, I like the idea of a dashboard, I think we’re gradually getting used to that. I think the dashboard is still in relation to where you’re at with other centres, or how you’re going compared with previously. So, whether you can have some trend line, also, as to whether it’s up or down compared with previous results, that might be useful.” P 14, metropolitan  “Then they can look at things in more segmented form. Yeah. Something like that might be easier. And then they can focus on what they need to focus on.” P 11, regional  “It’s very visual and I think if you were - if we were trying to explain where the data was to people that need to get that message across clearly, whether we’re pushing how well we’re doing and saying where all the green bits are or if we’re pushing an agenda to say we need to do better or we need more help and pushing the red parts of the dashboard, that would be helpful. I mean it is really, really easy to understand so I think that would be very helpful.’ P 5, metropolitan | 79% of total sites  66% of metropolitan  88% of regional |
| Domain: characteristics of intervention  Sub-domain: design quality and packaging  Option: data visualisation in a report card | “A report card may not be helpful in illustrating actionable clinical improvements” | “It, again, just seems like, “Oh yeah, they’re just telling us where we need to be headed.” I don’t think the format of that – because they are just statements. It might be okay as the front page of a report, as a summary in something, but in terms of individual staff, in a service, taking notice of it, I don’t think they’d look at it” P 12, regional  “I think the messaging has to be carefully worded because if it comes across as you’re not doing something right, it’s just going to be annoying for the clinicians because they’re all working hard to try and improve the situation. It needs to be worded very carefully. Like, needs work but the problem is it just depends on how much more work people can do!” P13, metropolitan  “I'm not sure how I look at that. It really depends how you stratify or sub stratify the data because again if [it does] go back to the traffic light, I might have red HbA1c and they may say, oh look, this is too low for comfort but for me I'm entirely comfortable with it.” P 9, metropolitan | 43% of total  50% of metropolitan,  38% of regional |
| Domain: characteristics of intervention  Sub-domain: design quality and packaging  Option: data visualisation in an individualised PowerPoint deck | “A pre-populated PowerPoint deck would be helpful for disseminating the data” | “I think a PowerPoint presentation would be great because we could do that as a team and like I said we don’t want a detailed report that details every item of the audit, we just want the key points basically.” P 8, metropolitan  “a PowerPoint presentation, it’s easier to present the data and generate conversation and feedback within the team about what could improve, how we can go about improving. Whether it’s service delivery or whether it’s a way of managing certain populations.” P 13, metropolitan  “It would make it easier for people like me that would do the presentation anyway…It would be great as a resource if you were going to present something at a conference…it’d be good to show the data.” P 11, regional | 100% of total sites |

**Table 3. Belief statements relevant to Aim 3**

| **Aim 3: To elicit user perceptions regarding the barriers to implementing feedback in its current format** | | | |
| --- | --- | --- | --- |
| **CFIR domain/ subdomain** | **Belief statement** | **Illustrative quote** | **Coverage** |
| Inner setting  Implementation climate  Compatibility | “There’s a lack of engagement with the audit because of clinical pressure” | “I think people, possibly, aren’t as invested in it as we would like them to be. Because, the way the audits occur we’re funding a nurse, who doesn’t normally work with those clients [to collect] the data, so they’re not really invested right from the start. Yeah, they know the audits are happening, they know it’s their clients that are being audited, but they’re not really involved in an active way, from the beginning. So, to them, I think, they just possibly see it as, “Oh, another thing I’ve got to add to my list of jobs to do.”” P 2, regional  “I don't have the luxury on my - because of the continual pressures in our department here, we have to discharge anyone who's vaguely looking at getting close to [HbA1c of] 7% because we've got tons of other patients on 10 or 9% or more who need to probably access us to see how we can help” P 9, metropolitan  “Often I come back and think, “Right there are things that we need to work on” but then it seems like I never get around to actually following up on it and I think part of the reason – because I have worked in larger centres before – is that you don’t have that team to get it up and running.” P 10, regional | 79% of total  88% of regional,  66% of metropolitan |
| Inner setting  Implementation climate  Relative priority  Topic: use of the report | “Reviewing ANDA feedback and utilising it for developing QI activities is a low priority” | “Well we would say that we technically probably haven’t sat down as a team and gone through it as such… One, because a time factor, and two, because we’ve never worked out how to utilise the document to – I don’t know – use to our advantage so to speak.” P 4, regional  “It’s not that people aren’t wanting to improve their practise, though, and it’s not that people don’t want the best quality service for their clients, that’s not it at all. It’s just yeah, everyone’s time poor, and it’s seen as an additional task that they’ve got to take the time to look at and invest in.” P 2, regional  “I’m telling you that just to demonstrate that among the clinicians in general, clinical and nursing, there’s not a great incentive for them to use this information.” P 6, regional | 57% of total  63% of regional,  50% of metropolitan |
| Inner setting  Implementation climate  - Readiness for implementation  Access to knowledge and information | “Not everyone knows how to use the feedback to inform QI activities” | “I think the report in itself, we know what’s going wrong. It’s actually having the means to address it and time is often a big issue, but sometimes it’s just the know-how. It’s like well what do we do? How do we go about it? Yeah, I think most people want to fix things, but it’s just well how do we change the same old same old? What are we missing?” P 10, regional  “I guess it’s - again and that may be where the webinar comes in where, again, that discussions around the webinar of how to use the report and where you produce the report in terms of not just ticking boxes, for quality improvement in annual accreditation - hospital or community health or GP accreditation process but also around future planning, strategic planning and increasing staffing or development of new programs. Again, and I know it’s stating the obvious but maybe for some people you’d have to actually [show] how to really use the report to improve services rather than just tick the box to say you’ve done it.”  P 5, metropolitan  “I think it’s been important to just make you aware of the importance of making those reports accessible to GPs. Because that’s who you’re really targeting. You’re not targeting me. I know what needs to be done. It’s them that need to do it. So, they need to be the focus of the reports, as much as me.” P 11, regional | 43% of total  50% of regional,  33% of metropolitan |
| Inner setting  Implementation climate  - Readiness for implementation  Available resources | “We have limited resources for implementation” | “The biggest one is time to be honest and I guess along with that is the resources. In any public hospital the focus is on service provision, the delivery of clinical care. Although QI obviously has a flow on effect for that if you’re out on the ward all the time or in the clinic all the time or don’t have the admin support or the IT support that is an obstacle to QI.” P 7, metropolitan  “It’s how they allocate resources. And the fact is, they have hardly got enough for their own needs, let alone to add extra on for something this rigorous. And it is incredibly rigorous in a practise like ours to do this.” P 11, regional  “We see patients who have other complex comorbidities, which makes their care even more intensive. So, I think, for those reasons, quality assurance and quality improvement programs, often are left as a designated research project rather than being something that’s, necessarily, part of the service. So, it’s lack of resources and time and increased complexity of patients, basically, and their care needs.” P 12, regional  “So, it’s getting the staff on board to accept that a change is required; to do that you need the data to show that there is an issue currently, that then needs to be addressed. And then it’s problem-solving, how are we going to address the issue?’ and to get the team to come up with those solutions, I guess, to see how we’re going to change it. And then the actual implementation.” P 14, metropolitan | 57% of total  63% of regional, 50% of metropolitan |
| Inner setting  Implementation climate  - Readiness for implementation  Leadership engagement | “Lack of leadership engagement is a barrier to implementation” | “…it could be useful if we had the right platform, and I mean like a committee or something that was dedicated to actually analysing the data and influencing some quality improvement activity. I have to take time to wade through the information, currently, and then present it in a form that management are, actually, going to take notice of.” P 2, regional  “Now, the director has said that they never act on ANDA results as it’s not discussed in detail at QI meetings.” P 6, regional  “I mean whether it's useful sometimes to sort of use benchmarking data to make an argument to the hospital executive that gee, your - look, my indicators are not so good here, we're trying our hardest, we don't have enough staff, that usually is not a conversation which generally goes very well anyway, when our very tightened budget, they basically be telling [us], oh well, just work with what you've got and change your processes to make it better, which is a challenge to us to always try and do.” P 9, metropolitan | 43% of total  37.5% of regional,  50% of metropolitan |

**Table 4. Belief statements relevant to Aim 4**:

| **Aim 4: To elicit user perceptions regarding the enablers to more effective use of feedback** | | | |
| --- | --- | --- | --- |
| **CFIR domain/subdomain** | **Belief statement** | **Illustrative quote** | **Coverage** |
| Inner setting  Implementation climate  - Readiness for implementation  Leadership engagement | “We can use the data in ANDA feedback to engage leadership” | “I think it’s actually because it is benchmarked that, really, time managers in our organisation take note. I would hate to see that removed from the audit and the report, because that’s really speaking executive management language, and it’s incredibly powerful, so just from a, I guess, purely a political point of view, I would just hate to not have that benchmarking. Because we really use that as leverage if we have the highest number of patients born overseas or we have the lowest uptake of something, that’s incredibly important for us.” P 5, metropolitan  “But I actually myself prefer hard copies, but it’s – attending health professionals is really useful, because most of ours are no to things that we know it’s obvious. They don’t see a social worker or a diabetes specialist, a psychologist because we don’t have those services readily available, and that’s useful to take to our higher beings and sort of say “well this is an issue”. So, it is useful. It’s really useful to have that sort of data to take when we’re seeking more services.” P 10, regional | 29% of total  37.55 of regional, 17% of metropolitan |
| Implementation process  Engaging  Champions | “Success stories and the experiences of other centres are enablers” | “But also having perhaps one of the organisations that are doing it well, or having a call out to those that are meeting the needs and how are they addressing it, how are they achieving that result?... there are little things like just being aware of where the services and how to access those services, especially in an ever-changing population, there are things that I think we can learn from other places that are doing it well.” P 10, regional  “it’s always good to hear what other people are doing and how they’re achieving it, and then you go through the mental process of ‘immediately, what are the barriers to sort of doing it?’ in your own centre, and then that’s where I guess you then want to be able to access the people who have implemented it, whether they came across those barriers and how they overcame them, to have that connection back to them, I think.” P 14, metropolitan  “What I would like support with, maybe, is in a presentation like that being able to say, “Well, some of our more exemplary primary care centres are doing these activities to reducing their HbA1cs, for example. So that, when I’m presenting to staff, I can say, “These are results, and this is what some of the organisations that have improved this index that was measured, this is what they’re doing. Maybe you could think about adopting some of those strategies.”” P 2, regional | 43% of total  38% of regional,  50% of metropolitan |
| Implementation process  Engaging  External Change Agents | “Mentoring from other centres would be an effective enabler of implementation” | “I’m sure there’s not only one centre that has the same issues. It would be good to work together and if there’s not a solution, work together and try and address it in the same way so that we can see those outcomes are working or – I hate this reinventing the wheel all the time, that round Australia five centres might have the same issue but we’re all trying to fix it with our own little committees and things rather than if someone’s got the answer, why not share it?” P 10, regional  “Then we wanted to change our model of care so I went and visited the XXXX which, as you know, is a centre of excellence. They weren’t at that stage, but they had this multidisciplinary team clinic model of care. We went along and we spent the day with them and it was really like having the blinkers taken off.” P 7, metropolitan  “I suppose NADC does do a fair bit of that in the various conferences or the annual conference. It was good to have a chat with how other diabetes centres are innovating in some areas, finding solutions for their problems in other areas and I think that was quite useful…” P 9, metropolitan  “I think clinicians in general become totally absorbed and overwhelmed in their face-to-face clinical work, unless there’s a lot of emphasis or guidance from someone like yourself, or a research program is focused on that, other than clinical outcomes.” P 6, regional  “But it would be good to also have one of the city hospitals sort of reach out and open the doors. Like for us to make contact, you go through, do a web search or whatever, but to actually get to the person that’s in those teams is quite a process. So it would be actually nice if annually they sent out a letter to their hospitals in their catchment and introduced themselves, gave us a fast track phone number or email address to contact and sort of welcomed questions and queries like this so that you weren’t spending time finding the right person going through hospital switchboards and the like…I think tertiary centres need to be aware that diabetes is not managed solely like it is in a tertiary centre.” P 6, regional | 43% of total  38% of regional,  50% of metropolitan |
| Implementation process  Engaging  External Change Agents | “Peer to peer support is an enabler” | “I went along and networking with other services and seeing all of these amazing QIs and amazing things that other centres were doing with less or more resources or less or more staff and also sharing some of the challenges that we all face, that was probably the first motivating factor.” P 7, metropolitan  “And being there as a support network for the smaller regions. If you’ve got patients that are leaving your centre and moving to a smaller one it’s…having the doors open to contact. Because I find the only way you network is if you’ve met people or know them before. You never get information from the big centres saying, “Here we are. You’re welcome to contact us.”” P 10, regional | 14% of total sites,  13% of regional,  17% of metropolitan |
| Outer setting  External Policy & Incentives | “External policy and incentives can be effective enablers” | “I guess the other thing too is reminding clinicians that it’s all part of our PD. Our colleges expect us to accumulate various [PD] points to achieve our accreditation each year and I always put ANDA down, and some good points on audit so it’s another way of getting buy-in from clinicians.” P 7, metropolitan  “When we last had our – not our last accreditation, but the one before that in 2014, the fact that we’d participated in ANDA was an incredibly important element of our quality improvement. We had a lot of evidence to show that we’d done good quality improvement.” P 11, regional  “So it’s – about enabling us to make more effective use of feedback. AHPRA, you know? The organisation we’re all required to be registered with? The medical board has decided that as part of continuing professional development to maintain registration, every physician has to be involved in measurement outcomes. So that is an enabler” P 6, regional | 43% of total sites,  50% of regional,  33% of metropolitan |
|  |  |  |  |

**Table 5. Participant perceptions regarding desired cointervention that is likely to support implementation of feedback and development of QI activities**

| **Aim: To elicit user perceptions regarding desired cointervention that is likely to support implementation of feedback and development of QI activities** | | |
| --- | --- | --- |
| **Concept** | **Illustrative quote** | **Coverage** |
| Coaching - yes | “Yeah, the thing is we are very time poor, but it could be good if someone could go this is where you’re doing well, this is what you need to improve on, I think that would be useful, but it would need to be straight to the point.”  P 8, metropolitan  “Yeah, that would be really good. We can take that out to our committees and things, yeah.”  P 4, regional | 29% of total,  17% of metropolitan,  38% of regional |
| Coaching - no | “I would hope that it was presented in a way that you could, easily – like you wouldn’t need coaching if it was clear how to interpret it. Yeah, I’m not sure about the value of needing to walk someone through the results.” P 2, regional  “I can’t envisage what you’d be able to add talking over the report, given that it’s just a set of data, unless people are misconstruing what the data means, because it’s fairly well explained in the report, itself, what the data means.” P 14, regional  “Gee, I think practically that would be hard. I’m just thinking of the other side of the equation; actually delivering on that would be a huge impost on your time. I’m not sure it would add a whole lot more to what we can glean from a report, that the gain from it I think is probably not going to be worth the effort.” P 14, metropolitan | 43% of total,  33% of metropolitan,  50% of regional |
| Webinar/ website exemplars of QI development | “So, if something’s been successful, share it. I know people do that at conferences and things, but not everyone can get to those, and it’s not specifically about this data. So, if there was some sort of showcase facility, on the website, that specifically related to quality improvement activities that can come from such a report, that would be useful.” P 12, regional  “I think possibly if there [were] more webinars around it where people could access around - again maybe a webinar for first timers using it, a webinar around some of the tricks of getting the best data and what have you, understanding the reports. I think if there [were] webinars that were available to look at any time for  members participating, I think that would be incredibly helpful.” P 5, metropolitan  “I'd like to get some ideas about perhaps how other institutions perhaps similar to mine with similar funding issues, similar problems, have tried to resolve the issues that they had… it would be probably something similar to a website, I suppose, where people can post something, this was the problem, this is how we solved it or tried to solve it and we actually found usefulness from that. So I think, yeah, it could be useful for us to get some ideas.” P 9, metropolitan  “So I was going to suggest that as part of your report, when you're looking at the areas that need improvement, you could do a QI example, like in case studies, let’s say of smoking, or cholesterols weren’t being measured.” P 6, regional  “But also having perhaps one of the organisations that are doing it well, or having a call out to those that are meeting the needs and how are they addressing it, how are they achieving that result?...that’s probably what it needs to be, more of a mentoring I guess from those that are doing well.” P 10, regional | 50 % of total  50% of metropolitan,  50% of regional |
